# Machine learning approaches for the prediction of soil aggregate stability

**DOI:** 10.1016/j.heliyon.2021.e06480

**Published:** 2021-03-13

**Authors:** Yassine Bouslihim, Aicha Rochdi, Namira El Amrani Paaza

**Affiliations:** Hassan First University, Faculty of Sciences and Techniques, Department of Applied Geology, Settat, Morocco

**Keywords:** Pedotransfer functions, Soil aggregate stability, Mean weight diameter, Multiple linear regression, Random forest, Remote sensing data

## Abstract

Currently, many Pedotransfer Functions (PTFs) are being developed to predict certain soil properties worldwide, especially for difficult and time-consuming parameters to measure. However, very few studies have been done to assess the feasibility of using PTFs (regression or machine learning methods) for predicting soil aggregate stability. Also, the Random Forest (RF) method has never been used before to predict this parameter, and no study was found concerning the use of PTFs methods to estimate soil parameters in Morocco. Therefore, the current study was conducted in the three watersheds of Settat- Ben Ahmed Plateau, located in the center of Morocco and covering approximately 1000 km^2^. The purpose of this study is to compare the capabilities of the machine learning technique (Random Forest) and Multiple Linear Regression (MLR) to predict the Mean Weight Diameter (MWD) as an index of soil aggregate stability using soil properties from two sources data sets and remote sensing data. The performance of the models was evaluated using a 10-fold cross-validation procedure. The results achieved were acceptable in predicting soil aggregate stability and similar for both models. Thus, the addition of remote sensing indices to soil properties does not improve models. Results also show that organic matter is the most relevant variable for predicting soil aggregate stability for both models. The developed models can be used to predict the soil aggregate stability in this region and avoid waste of time and money deployed for analyses. However, we recommend using the largest and most uniform possible data set to achieve more accurate results.

## 1 Introduction

Soil is a natural resource of public interest that is under increasing environmental pressure and, therefore, must be sustainably managed for the benefit of future generations. This management cannot be reached without a proper understanding of the different soil characteristics and properties. Aggregate stability is one of the essential factors in soil conservation and maintenance of its environmental functions (Hanke and Dick, 2017), it affects water (Kunhikrishnan et al., 2012), and store and stabilize organic carbon (Kodešová et al., 2008). Furthermore, an increase in soil structural stability can directly increase the resistance against erosive agents and compaction (Chaplot and Cooper, 2015). Stable soil aggregates form a stable soil structure, allowing optimum movement and storage of gases, water and nutrients (Gliński et al., 2011). All this information could confirm that soil aggregate stability may be a useful indicator for monitoring soil quality (Chaplot and Cooper, 2015).

Soil aggregate stability can be measured with many different methods, which have been the subject of several reviews (Amezketa, 1999; Le Bissonnais, 2016; Nimmo and Perkins, 2002). According to Jastrow and Miller (1991), this diversification of measurement methods can be explained by three reasons: (1) the existence of different mechanisms that produce destabilization, (2) the different scales at which stability can be determined, and (3) methodological reasons.

More recently, the most common method used for aggregate stability measurement is Le Bissonnais's method, which has become established as the standard approach to determine the soil's aggregate stability. This method has been adopted as the international standard with the award of the (ISO/FDIS 10930, 2012). Despite the consensus on this measurement methodology, it remains difficult to apply routinely since it is very time-consuming. Indeed, one needs to deal with three different tests, including fast wetting (FW), slow wetting (SW) and mechanical breakdown (WS), repeated three times for each analysis, and a large quantity of ethanol would be necessary for this method (Le Bissonnais, 2016). Generally, it is a common problem for all other soil properties, especially when talking about a large surface and large samples to be analyzed.

To overcome this problem, scientists have searched for alternative solutions. Therefore, Pedotransfer Functions (PTFs) have appeared to be the best solution. These approaches are used to estimate soil properties by easily measurable soil parameters (Gunarathna et al., 2019). It can also be defined as predictive functions of certain soil properties from others easily, routinely, or cheaply measured properties. The most readily available data come from soil surveys, such as field morphology, texture, structure, and pH (Odeh and McBratney, 2005).

During the last few decades, regression methods have been widely used to develop PTFs worldwide. Recently, machine learning methods have been deployed in PTFs development, such as the K-Nearest Neighbor (KNN) (Mihalikova et al., 2014), Cubist (Kuhn et al., 2013), Artificial Neural Networks (ANN) (D'Emilio et al., 2018), and Random Forests (RF) approaches (Dharumarajan et al., 2017). Despite those frequent applications, machine learning approaches remain hardly used to develop PTFs.

The possibility of using PTFs methods to estimate the different soil parameters has been widely studied all around the world, especially for parameters that are difficult and time-consuming to measure, such as soil plasticity (Al Masmoudi et al., 2021), soil carbon (Keskin et al., 2019), bulk density (Souza et al., 2016), soil water content (Santra et al., 2018), hydraulic conductivity (Zhao et al., 2016), soil phosphorus (Valadares et al., 2017), soil nitrogen (Dessureault-Rompré et al., 2015) and total silicon concentrations (Landre et al., 2018). On the other hand, very few studies have been done to assess the feasibility of using PTFs (regression or machine learning methods) for predicting soil aggregate stability (Annabi et al., 2017; Besalatpour et al., 2013; Marashi et al., 2017; Melo et al., 2018). Following this research, we have seen that the Random Forest method has never been used before predicting soil aggregate stability. Based on our literature review, no study was found concerning the use of PTFs methods to estimate soil parameters in Morocco.

This study's objectives were to compare the capabilities of Multiple Linear Regression (MLR) and Random Forest (RF) to derive PTFs between soil aggregate stability and different sets of input variables. The developed PTFs can be used to predict the soil aggregate stability in this region and avoid waste of time and money deployed for analyses.

## 2 Materials and methods

### 2.1 Study area

**Figure 1:**
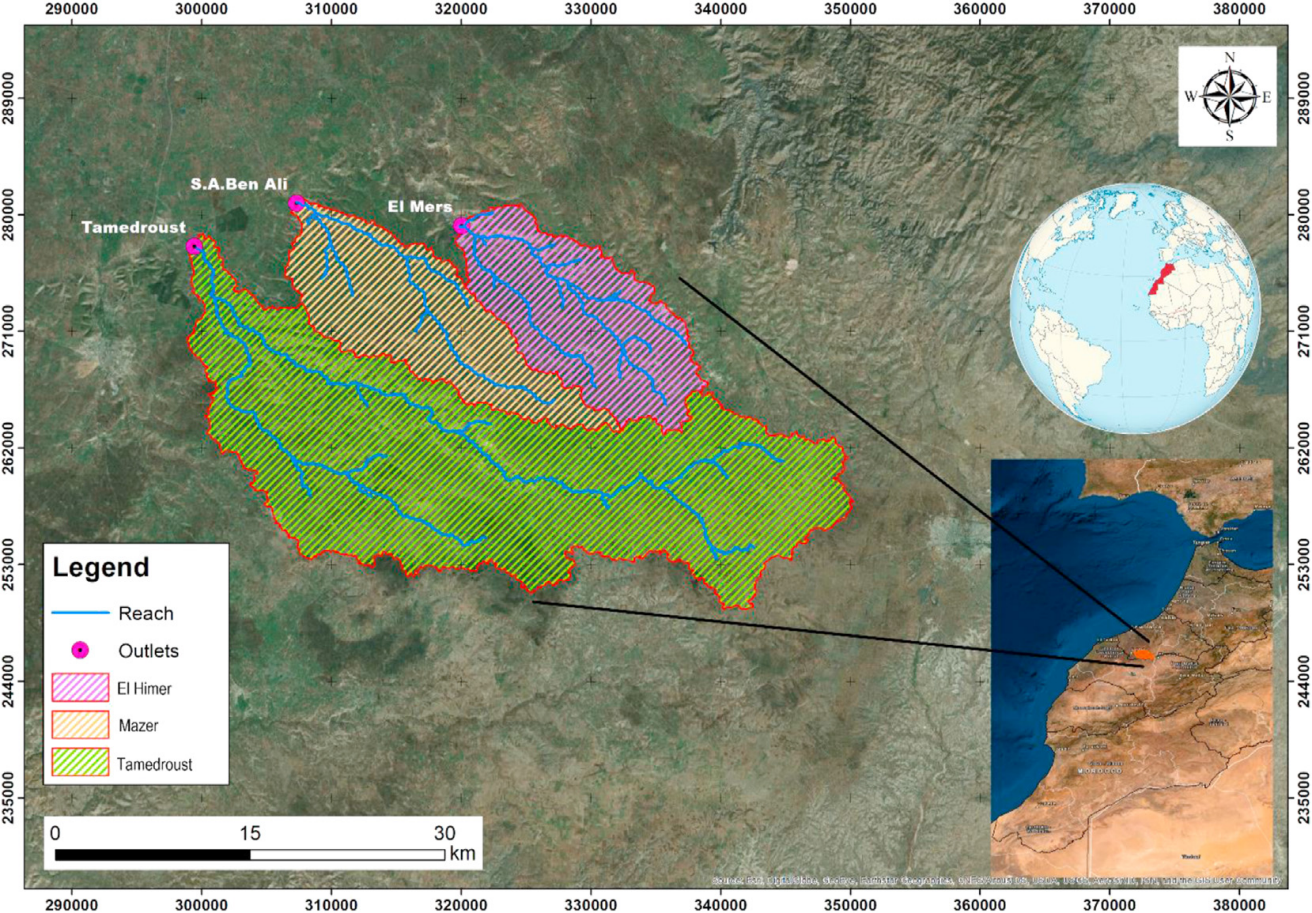
Location of the study area in the region of Chaouia Ourdigha (Settat-Ben Ahmed plateau, Morocco).

This study was conducted in Chaouia Ourdigha, precisely in the three watersheds of Settat Ben Ahmed Plateau (Figure 1). Located in the center of Morocco and covering a total area of approximately 1000 km^2^, the Tamedroust watershed covers more than half of the total area (642.42 km^2^). While the Mazer and El Himer watersheds occupy 179.2 and 177.7 km^2^, respectively. The area and all other physical characteristics of the three watersheds were determined from the DEM (Digital Elevation Model) and the GIS program (ArcGIS). The climate in this region is semi-arid, with an average annual rainfall of 298 mm/year. The most humid period spreads over four months, from November to February. Moreover, the months of June to August are often completely dry (Bouslihim et al., 2019). The mean annual temperature in the study area is approximately 17 °C.

The study area is part of the phosphate plateau, and the most representative soil type is calcisols. The three watersheds are poorly covered with vegetation.

### 2.2 Prediction models

For comparative assessment, two different methods were used to analyze the feasibility of using the PTFs techniques to predict the soil aggregate stability from routinely measured soil properties and remote sensing indices.

Multiple Linear Regression (Bottenberg and Ward, 1963) is one of the prediction methods and a widely known modeling technique. Linear Regression establishes a relationship between the dependent variable (*y*) and one or more independent variables (*x*) using a best fit straight line. It is represented by the following Eq. (1) (Marashi et al., 2017):

(1) $$y=b_{0}+\;b_{1}x_{i,1}+\;b_{2}x_{i,2}+\ldots +\;b_{k}x_{i,k}+\;e_{i}$$

where *y*_*i*_ is the dependent variable, *b*_*0*_ is a constant (the intercept), *x*_*i,k*_ is an independent variable, *b*_*k*_ is the vector of regression coefficients called slope, and *e*_*i*_ represents residuals not explained by the model.

The second model used in this study is the Random Forest; it is a flexible and easy-to-use machine learning algorithm developed mainly to overcome the single regression tree limitations (Breiman, 2001). During the model's construction, many regression trees are grown with randomly selected combinations of input variables which gives many different results and the final prediction is achieved through voting (Anysz et al., 2020). In this way, the model will be more robust to outliers and noise than a single regression tree. Prediction is based on a whole set of regression trees, while the results of all individual trees are averaged, or weighted average is calculated (Van Looy et al., 2017). Random Forest modeling can improve predictions made by classification and regression trees (Breiman, 2001). Two important parameters in the RF method are the number of trees (*ntree*) and the number of variables available for selection in each split (*mtry*) (Houborg and McCabe, 2018). The model was performed using the Statistical Package for Social Sciences (SPSS) software (version 25.0).

### 2.3 Soil properties and remote sensing parameters

**Figure 2:**
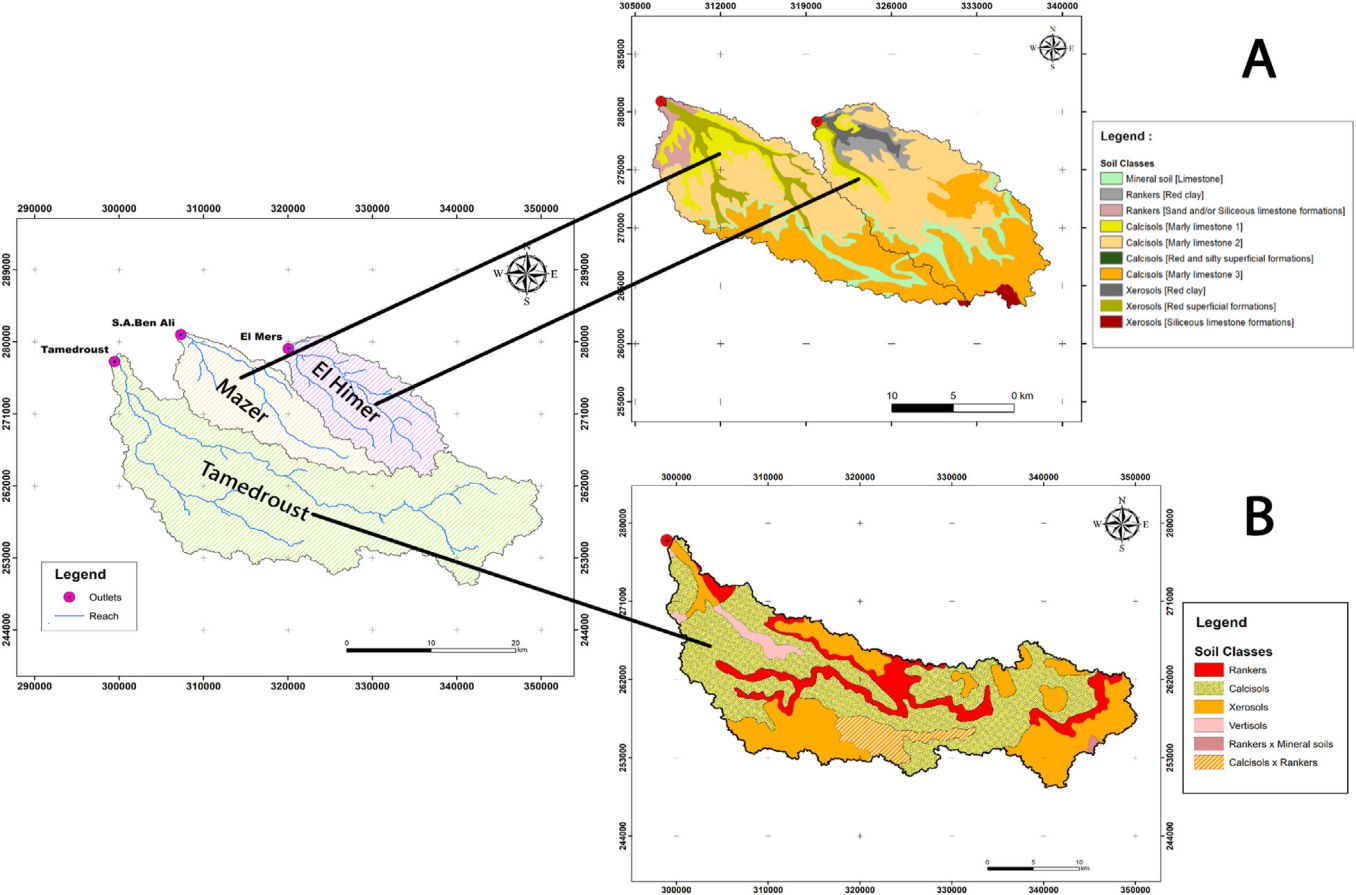
Soil classes map of A: Mazer and El Himer watersheds and B; Tamedroust watershed.

The study area is covered by two soil maps with a different scale. The first soil map was obtained from the pedological study by the Ministry of Agriculture and the Hassan II Agricultural and Veterinary Institute (IAV) in 1985 at ascale of 1:100000 (Figure 2 A). This map covers the Mazer and El Himer watersheds and a small part of the Tamedroust watershed. For this reason, we used a second map with a scale of 1:500000 realized by INRA-Morocco (National Institute for Agronomic Research), DMN-Morocco (National Direction of Meteorology) ICARDA (The International Center for Agricultural Research in the Dry Areas) and IDRC-CANADA (The International Development Research Centre-Canada) (El Oumri et al., 1995), which covers the whole area of the Tamedroust watershed (Figure 2 B).

**Figure 3:**
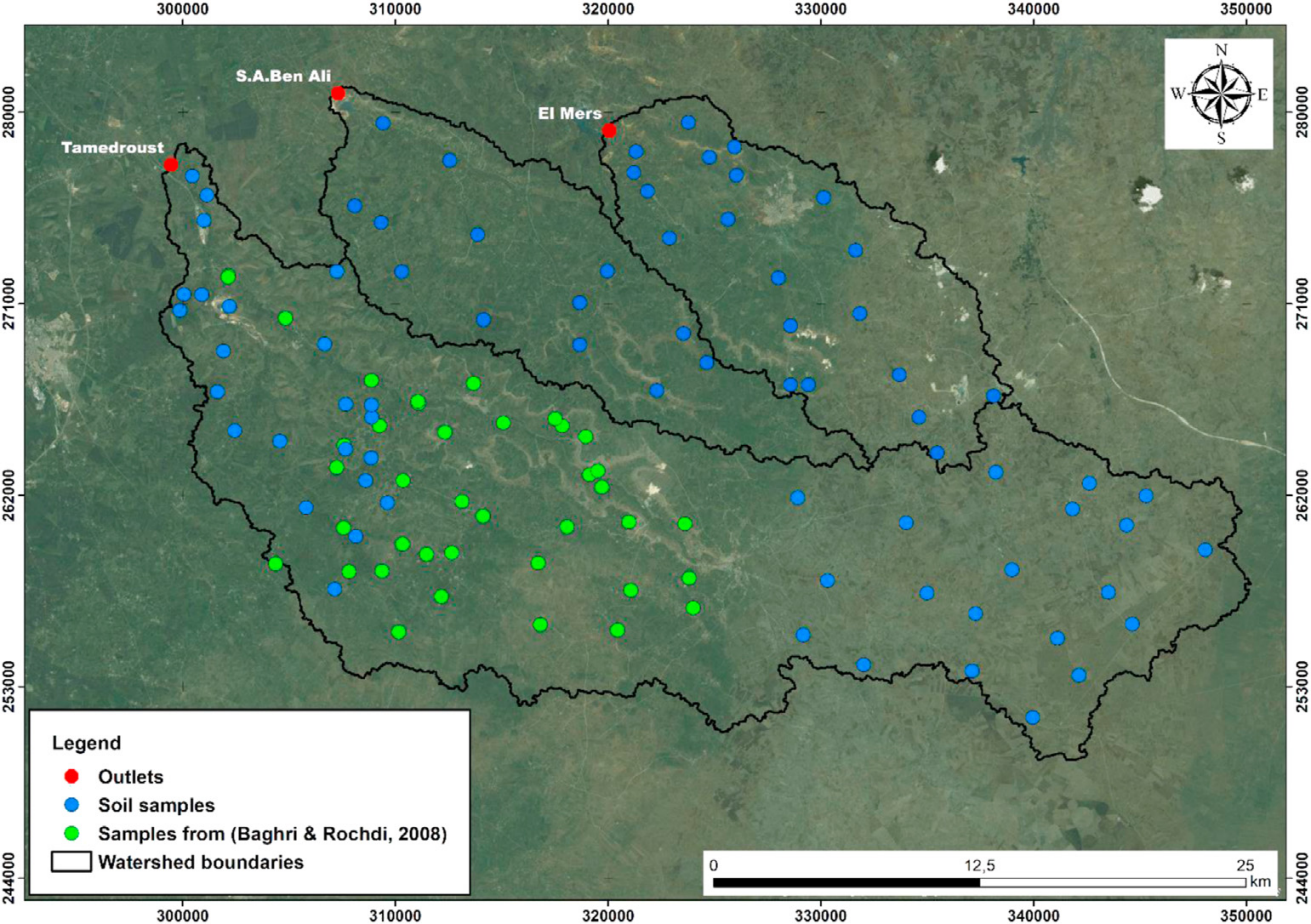
Location of the sampling points.

For soil analysis, a total of 77 soil samples were collected (0–40 cm depth) to cover the majority of the study area's surface (Figure 3) by taking two types of soil (disturbed and undisturbed soil samples). The undisturbed soil samples were taken by Kopecky rings in standard sharpened steel 100-cm^3^ to determine the bulk density (Dirksen, 1999). Moreover, all samples were analyzed for soil aggregate stability and their physicochemical properties.

All soil analyses were carried out at Hydrology and Soils Laboratory, Faculty of Science and Technology, Hassan First University (Settat, Morocco), following the standard operating procedures during all analyses.

Soil carbon was determined by the Walkley and Black procedure (Walkley and Black, 1934). This method is based on the oxidation of organic matter by potassium dichromate (K_2_Cr_2_O_7_) in the presence of sulfuric acid (H_2_SO_4_). The percent of soil organic matter (OM) was obtained by multiplying percent soil organic carbon by a factor of 1.724 following the assumption that organic matter is composed of 58% of carbon (Sleutel et al., 2007). Soil texture was determined with the standard French method, Sedimentation-Pipette method (NFX31–107). At first, we remove organic matter from all soils by using hydrogen peroxide. The finest particles (clay and silt) were determined with the Robinson pipette method. The sand fraction was separated via sieving at 50 μm. Soil bulk density (BD) was determined from the undisturbed core sampling method after drying the soil samples in an oven at 105 °C to constant weights. Soil pH was measured in water (pH water) using the Hanna pH meter. Furthermore, the Cation Exchange Capacity (CEC) was obtained from the ISRIC database (Batjes et al., 2017), and the Available Water Capacity (AWC) was calculated using (Saxton and Rawls, 2006) equations.

Aggregate stability was measured using the standardized method (ISO/FDIS 10930, 2012), noted in Le Bissonnais (2016). The air-dried soil was sieved of 5-mm mesh, and the 3–5-mm aggregates were selected for the three treatments: fast wetting, slow wetting, and mechanical breakdown by shaking after pre-wetting. Before the three treatments, aggregates were dried in the oven at 40 °C for 24 h to ensure that they are at a constant matric potential. The aggregate stability for each treatment was expressed by the mean weight diameter (MWD), which is the sum of the mass fraction of soil remaining on each sieve after sieving multiplied by the adjacent mesh's mean aperture. According to Le Bissonnais (2016), the calculated MWDs values were used to classify our soils into five classes (Table 1).

**Table 1:** Stability classes according to MWD values measured with the three treatments.

Class	MWD value/mm	stability
1	<0.4	Very unstable
2	0.4–0.8	Unstable
3	0.8–1.3	Medium
4	1.3–2.0	Stable
5	>2	Very stable

All remote sensing parameters were extracted from the imagery satellite (Landsat-8) using remote sensing techniques to derive these indices; their description and calculation formulae are presented in Table 2.

**Table 2:** Different indices (remote sensing parameters) evaluated in this research paper.

Index	Description	Equation	Reference
LAI	Leaf Area Index	3.618∗EVI−0.118	Boegh et al. (2002)
EVI	Enhanced Vegetation Index	2.5∗(ρPIR−ρRρPIR+C1ρR−C2ρB+L)[C1 = 6, C2 = 7.5, L = 1]	Huete et al. (1999)
GSI	Grain Size Index	GSI=(R−B)(R+B+G)	Xiao et al. (2006)
SAVI	Soil Adjusted Vegetation Index	ρPIR−ρRρPIR+ρR+0.5∗(1.5)	Huete (1988)
GVI	Green Vegetation Index	(−0.2848∗ρaero)+(−0.2435∗ρB)+(−0.5436∗ρG)+(−0.7243∗ρR)+(−0.0840∗ρMIR1)+(−0.1800∗ρMIR2)	Kauth and Thomas (1976)
BI	Brightness Index	$\sqrt{\rho G^{2}+\rho R^{2}}$	Khan et al. (2005)
RI	Redness Index	ρR2ρG3	Pouget et al. (1990)
SI	Salinity Index	$\sqrt{\rho G*\rho R}$	Dehni and Lounis (2012)
NDWI	Normalized Difference Water Index	ρPIR−ρMIRρPIR−ρMIR	Gao (1996)
MSI	Moisture Stress Index	ρMIRρPIR	Hunt and Rock (1989)
RVI	Ratio Vegetation Index	ρPIRρR	Kriegler et al. (1969)
DVI	Difference Vegetation Index	ρPIR−ρR	Bacour et al. (2006)
NDVI	Normalized Difference Vegetation Index	ρPIR−ρRρPIR+ρR	Rouse et al. (1973)
TNDVI	Transformed Normalized Difference Vegetation Index	0.5+((ρNIR−ρR)(ρNIR+ρR))	Bannari et al. (2002)

**Figure 4:**
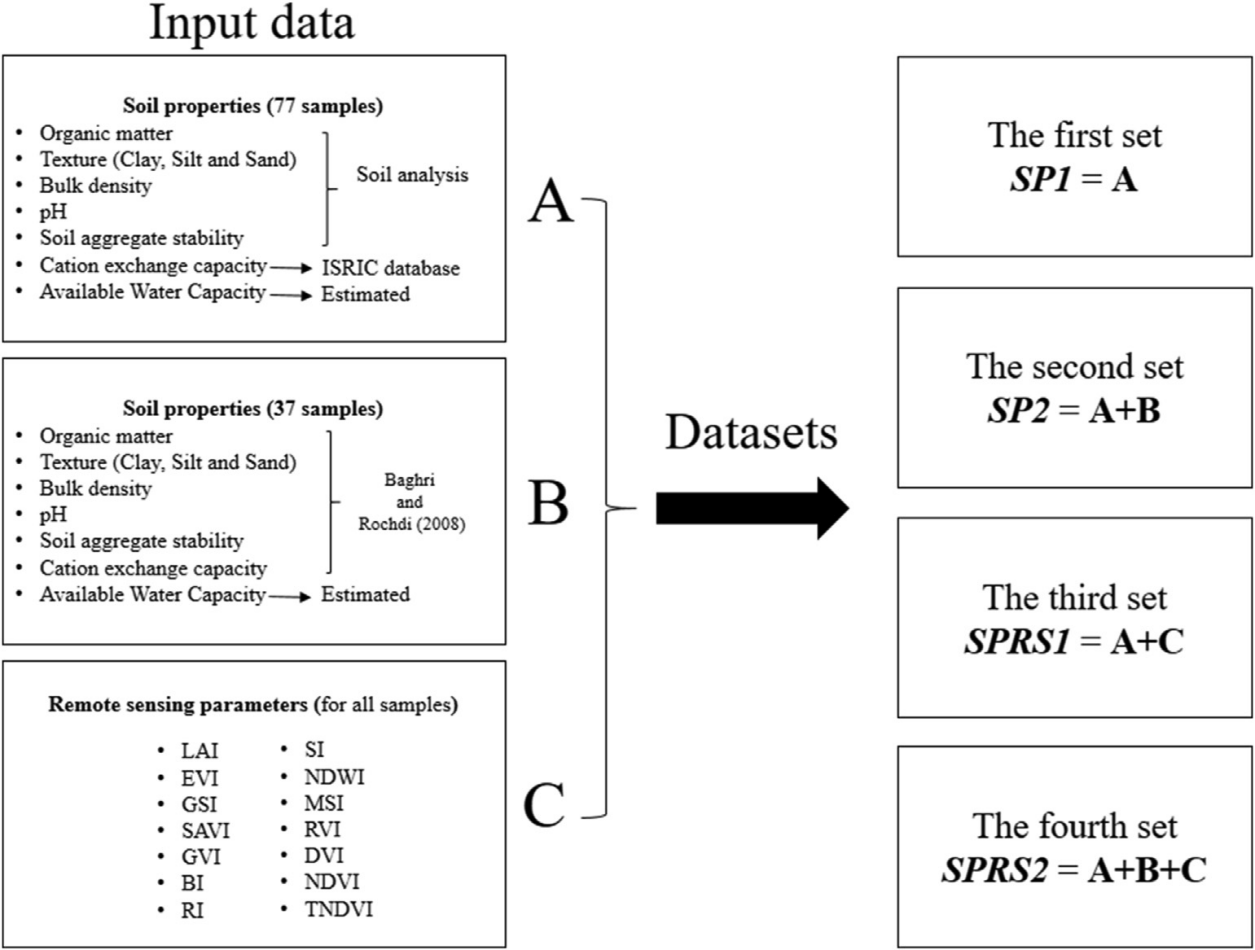
Soil input data used for the development of different models. (SP1: Soil Properties for 77 samples; SP2: Soil Properties for 114 samples; SPRS1: Soil Properties & Remote Sensing for 77 samples; SPRS2: Soil Properties Remote Sensing for 114 samples; LAI: Leaf Area Index; EVI: Enhanced Vegetation Index; GSI: Grain Size Index; SAVI: Soil Adjusted Vegetation Index; GVI: Green Vegetation Index; BI: Brightness Index; RI: Redness Index; SI: Salinity Index; NDWI: Normalized Difference Water Index; MSI: Moisture Stress Index; RVI: Ratio Vegetation Index; DVI: Difference Vegetation Index; NDVI: Normalized Difference Vegetation Index; TNDVI: Transformed Normalized Difference Vegetation Index).

Thirty-seven additional samples (BR08) were obtained from Baghri and Rochdi (2008) study to expand our database; these samples are located in the middle part of the Tamedroust watershed, as shown in Figure 3. The soil aggregate stability data obtained from this study were analyzed with a different method. For this reason, we have compared four different data sets (SP1, SP2, SPRS1 and SPRS2) to verify and avoid any influence of Baghri and Rochdi (2008) data. Figure 4 illustrates how the data was packaged to form the four sets.

• The first set (denoted as ***SP1***) consisted of soil properties alone for the first 77 soil samples.
• The second set (denoted as ***SP2***) included all soil samples (77 + 37)
• The third set (denoted as ***SPRS1***) combines soil properties and remote sensing indices for the first 77 soil.
• The fourth set (denoted as ***SPRS2***) included all soil samples (77 + 37) and all other remote sensing indices.

### 2.4 Evaluation of prediction accuracy

The MLR and RF models' performance was evaluated using a 10-fold cross-validation procedure that involved comparisons between the predicted and observed MWD values. Cross-validation provides a modeling structure for dividing several calibrations and validation sets, which guarantees that each sample can be assigned to the validation at least once. The greatest advantage of this method is that it runs reliably and is unbiased for a small sample set (Y. Hong et al., 2020). The created PTFs were also assessed based on the differences between the observed and predicted MWD, using two parameters, the coefficient of determination (R^2^) (Eq. (2)) and the root mean square error (RMSE) (Eq. (3)). Thus, we applied the model performance classification criteria defined by Li et al. (2016) as values of R^2^ < 0.5 (unacceptable prediction capacity), 0.5 ≤ R^2^ < 0.75 (acceptable prediction capacity), and R^2^ ≥ 0.75 (good prediction capacity), to evaluate model performance based on R^2^.

(2) $$R^{2}=1-\frac{\sum_{i=1}^{n}{\left(O_{i}-\;P_{i}\right)}^{2}}{\sum_{i=1}^{n}{\left(O_{i}-\;\bar{O}\right)}^{2}}$$

(3) RMSE=∑i=1n[Oi−Pi]²n

where *O*_*i*_, *P*_*i*_ and $\bar{O}$ are the observed, predicted and mean *O*_*i*_ value at site *i*, respectively, and n is the number of samples.

## 3 Results and discussion

### 3.1 Descriptive statistics of soil properties

Statistical analysis was performed on the whole data set (n = 114 samples) for different soil properties (pH, OM, clay, silt, sand, BD, CEC and AWC) and remote sensing indices (LAI, GSI, EVI, SAVI, GVI, BI, RI, SI, NDWI, MSI, RVI, DVI, NDVI and TNDVI). The descriptive statistics such as max, min, standard deviation, skewness and kurtosis) are shown in Table 3.

**Table 3:** Summary statistics of soil properties and remote sensing indices for the 114 samples.

Parameter	Min	Max	Mean	Standard deviation	Skewness	Kurtosis
pH	7.150	9.140	7.980	0.351	0.078	0.263
OM	0.287	6.693	3.765	1.395	-0.200	-0.213
Clay (%)	3.019	65.445	30.308	12.475	0.412	0.396
Silt (%)	3.484	66.920	32.112	13.876	0.427	-0.277
Sand (%)	5.240	93.497	35.495	14.706	0.614	1.216
BD	0.945	1.686	1.380	0.162	-0.424	-0.886
CEC	8.369	57.848	28.449	6.320	1.271	5.081
AWC	0.037	0.187	0.129	0.022	-0.356	2.501
MWDmean	0.477	2.975	1.595	0.481	0.219	-0.108
LAI	0.602	2.415	1.111	0.331	1.293	2.236
GSI	-0.076	0.148	0.065	0.038	-1.125	2.079
EVI	0.199	0.700	0.340	0.091	1.293	2.236
SAVI	0.167	0.490	0.269	0.061	0.994	1.148
GVI	0.098	0.188	0.143	0.017	-0.188	-0.139
BI	0.136	0.246	0.180	0.019	0.681	1.404
RI	7.173	15.016	10.511	1.328	0.030	0.545
SI	0.073	0.252	0.159	0.033	0.199	0.606
NDWI	-0.119	0.381	0.067	0.108	0.313	-0.520
MSI	0.448	1.271	0.894	0.189	0.058	-0.848
RVI	1.616	4.916	2.231	0.526	2.114	6.634
DVI	0.106	0.322	0.176	0.041	1.000	1.114
NDVI	0.235	0.662	0.368	0.084	0.908	0.833
TNDVI	0.858	1.078	0.931	0.044	0.778	0.475

BD: bulk density, CEC: cation exchange capacity, AWC: available Water capacity; MWD: mean weight diameter; LAI: Leaf Area Index; EVI: Enhanced Vegetation Index; GSI: Grain Size Index; SAVI: Soil Adjusted Vegetation Index; GVI: Green Vegetation Index; BI: Brightness Index; RI: Redness Index; SI: Salinity Index; NDWI: Normalized Difference Water Index; MSI: Moisture Stress Index; RVI: Ratio Vegetation Index; DVI: Difference Vegetation Index; NDVI: Normalized Difference Vegetation Index and TNDVI: Transformed Normalized Difference Vegetation Index).

Considering the whole data set (n = 114), soil properties showed significant variability over the study area. Soil pH ranged from 7.15 to 9.14 with a mean of 7.98 ± 0.351, and OM had a mean of 3.765 ± 1.395 with a value of min and max being 0.287 and 6.693, respectively. The range of the values of the coefficients of skewness varied from -0.424 to 0.219 (for pH, OM, BD and AWC), which indicates that most of the parameters are fairly symmetrical (skewness between -0.5 and 0.5), as confirmed by the coefficients of kurtosis, which have the same tendency.

In general, it can be said that most data distributions tend to be normal (except CEC). Hence, the mean value of each data set can be considered as the center of distribution (Nielsen and Wendroth, 2003). The high positive value of skewness coefficients for CEC (+1.271) indicates that the data are highly skewed. Also, the high values of kurtosis for CEC (5.081) and AWC (2.501) were probably due to the presence of one or more outliers (Brys et al., 2003).

**Figure 5:**
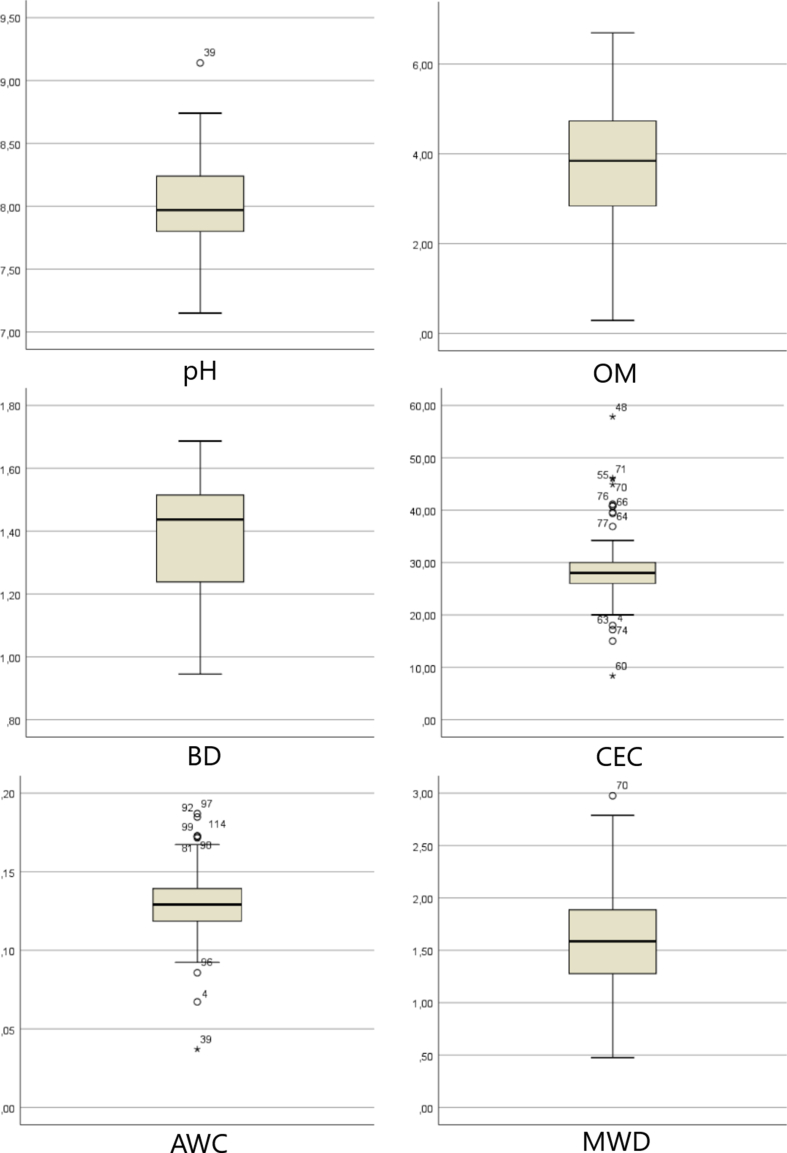
Box plots of different soil properties for the 114 soil samples. (pH: potential of hydrogen, OM: organic matter, BD: bulk density, CEC: cation exchange capacity, AWC: available Water capacity and MWD: mean weight diameter).

As can be noted in the box plots of all parameters (Figure 5), several values can be identified as outliers, especially at CEC and AWC, confirming earlier kurtosis results.

Clay fraction ranged from 3.019 to 65.445, with a mean and standard deviation of 30.308 and 12.475, respectively. Silt fraction ranged from 3.484 to 66.92, with a mean and standard deviation of 32.112 and 13.876, respectively. Sand fraction ranged from 5.24 to 93.497, with a mean and standard deviation of 35.495 and 14.706, respectively.

**Figure 6:**
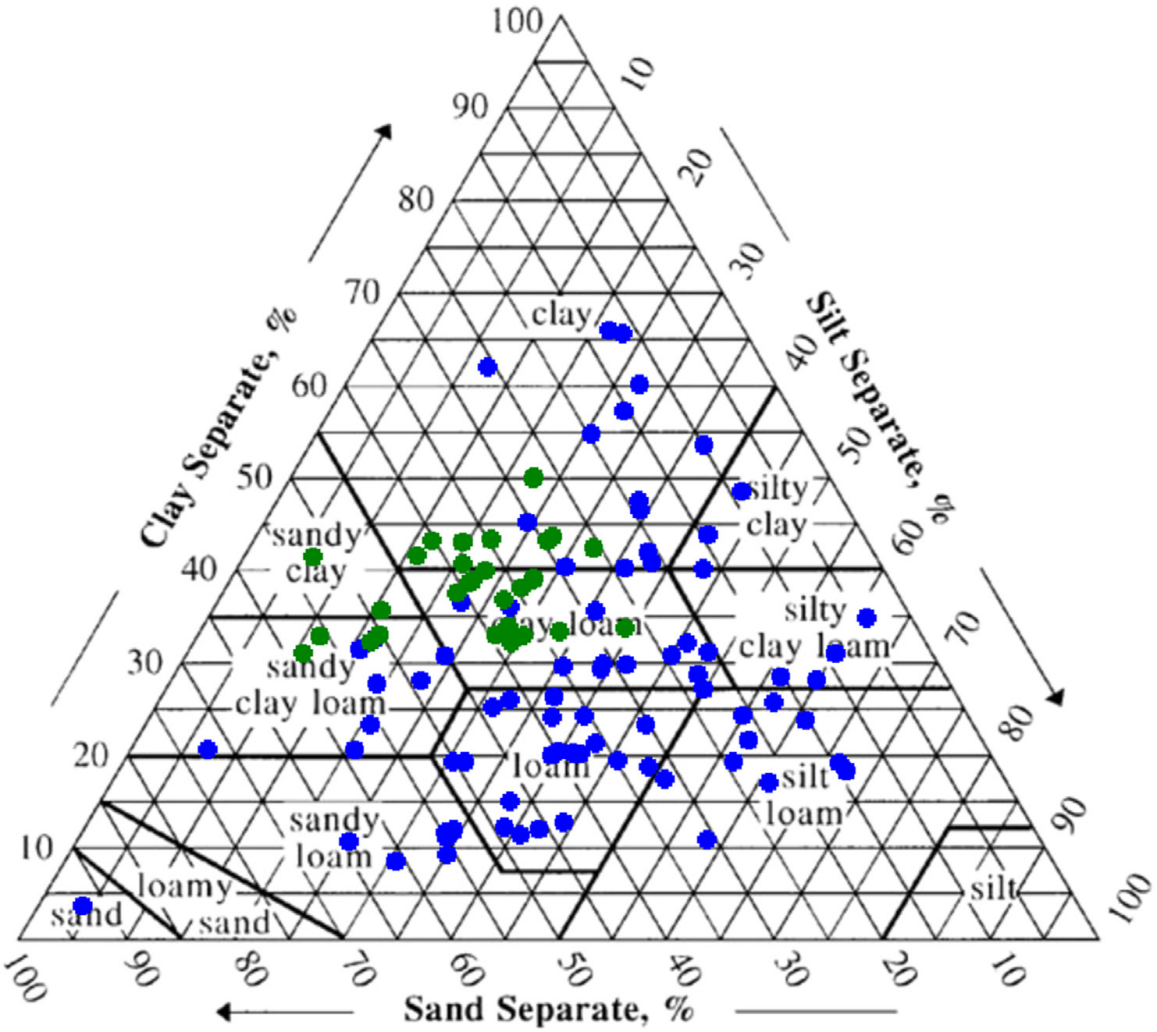
Distribution of soil samples (n = 117) inside the USDA soil texture triangle (Blue: SP1 data set 77 samples, Green: BR08 data set 37samples).

Different samples' textural Class was determined by referencing values for %Sand, %Silt and %Clay on the textural triangle. Figure 6 shows considerable variability in soil texture. It is generally due to the high spatial variability of soil in the three watersheds.

**Figure 7:**
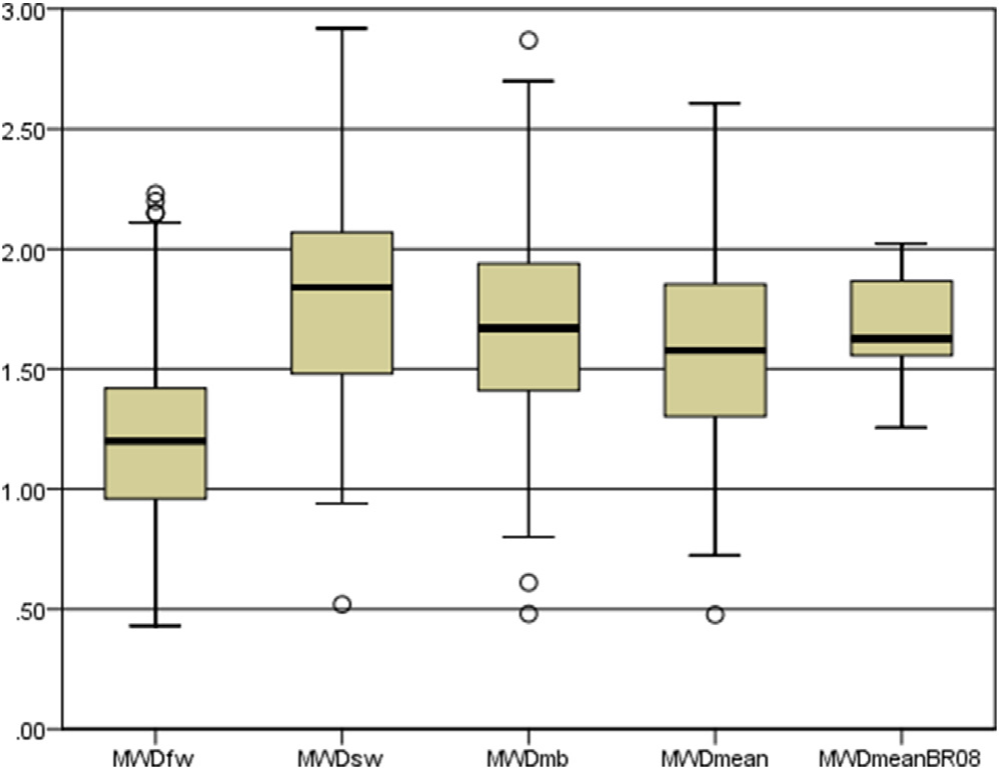
Distribution of Mean Weight Diameter (MWD) for 77 samples under (fast wetting: fw, slow wetting: sw, mechanical breakdown: mb, and the mean of the three tests: MWDmean) and MWD for the 37 samples (MWDmeanBR08).

The soil aggregate stability data of the 77 samples presented in Figure 7 show that the three indices can be classified in the following order: MWDsw (slow wetting) > MWDmb (mechanical breakdown) > MWDfw (fast wetting), which corresponds with the results of previous studies (Annabi et al., 2017; Chenu et al., 2000).

MWDfw had a lower value and varied between 0.43 mm and 2.23 mm with a mean of 1.225 ± 0.44 mm. It is caused probably by the rapid water penetration into the soil aggregate, which causes further slaking due to the pressure produced (Annabi et al., 2017).

MWDsw ranged between 0.52 mm and 2.92 mm with a mean of 1.8 ± 0.45 mm. Therefore, the MWDsw value was higher than MWDfw because slaking was reduced due to soil aggregate's slow wetting. For the last test, the MWDmb value was between MWDfw and MWDsw values. In this test, slaking does not occur because aggregate porosity is saturated with ethanol, which decreases the surface tension and contact angle (Annabi et al., 2017). Thus, the primary cause of the aggregate breakdown is due to the agitation and abrasion between aggregates (Le Bissonnais and Le Souder, 1995) MWDmb had a mean of 1.685 ± 0.47 mm with minimum and maximum value of 0.48 mm and 2.87 mm, respectively.

The MWDmean can provide an overall view of aggregate stability at different conditions in the field. MWDmean values indicate that soil aggregate stability shows significant variability and ranged from 0.47 mm to 2.6 mm, with an average of 1.57 ± 0.43 mm. For the whole data sets (114 samples), MWD is ranged from 0.477 to 2.975 with a mean and standard deviation of 1.595 and 0.481, respectively.

According to the classification proposed by Le Bissonnais (2016) (Table 1), no soil was classified as very unstable (<0.4 mm). The majority of the samples (63%) were classified as stable (1.3–2.0 mm), 20% of samples were classified as medium (0.8–1.3 mm), 13% of samples are very stable (>2 mm) and the rest of the samples (4%) were classified as unstable (0.4–0.8 mm). Therefore, a significant correlation was observed between the MWDmean and the three tests (MWDfw, MWDsw and MWDmb).

### 3.2 Multiple linear regression model performance

A high correlation between variables may influence the achievement of the expected results for the MLR. This is referred to as multicollinearity (between more than two variables) or collinearity (between two variables) (Kumari, 2008), which can cause unstable estimates of regression coefficients in linear and logistic regression models, incorrect variance estimates for the coefficients of those parameters in regression models, and some difficulties in the numerical calculations involved in fitting the regression model (Dohoo et al., 1997). Multicollinearity occurs in a data set due to the correlation between the predictors. Models derived from such data without a check on multicollinearity may lead to erroneous system analysis (Garg and Tai, 2013). This problem can be avoided by selecting the appropriate predictors from the data set and eliminate the variables that could affect the model results.

**Figure 8:**
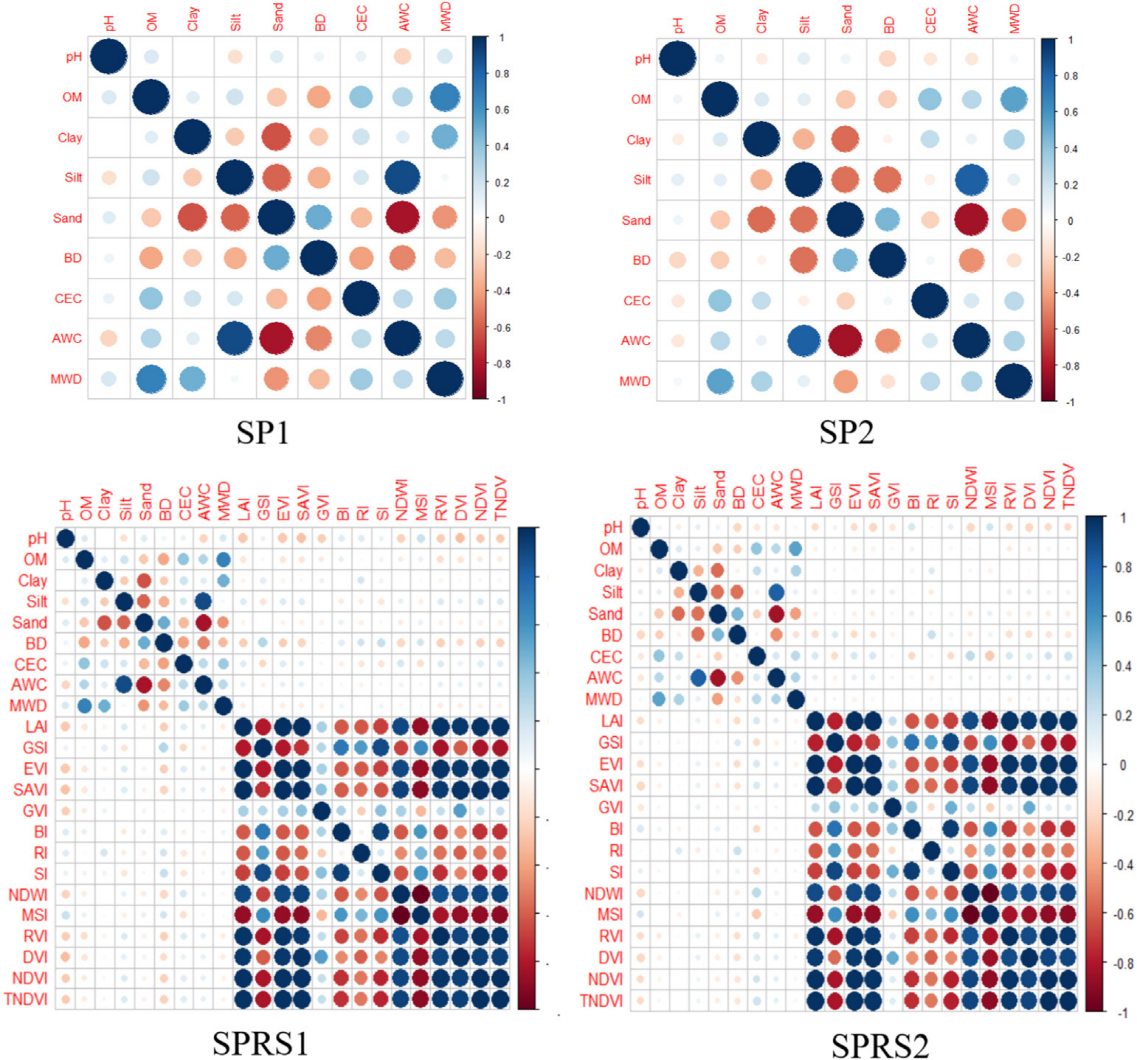
Correlation matrix between variables of different data sets. (SP1: Soil Properties for 77 samples; SP2: Soil Properties for 114 samples; SPRS1: Soil Properties & Remote Sensing indices for 77 samples and SPRS2: Soil Properties & Remote Sensing indices for 114 samples).

For this reason, the correlation was checked using the matrix of Pearson's between all independent variables of the four data sets (Figure 8). All correlations matrices were performed using the corrplot package in R (Wei et al., 2017).

For SP1 data set, sand and AWC were excluded from the list of input variables because of multicollinearity between clay/silt and sand, and the collinearity between silt and AWC.

For SP2 data set, the same variables detected in the SP1 data set were eliminated (sand and AWC), with the addition of BD because of collinearity with silt.

For SPRS1 data set and due to multicollinearity between remote sensing indices, we kept only NDVI and GVI. However, all other remote sensing indices were excluded without forgetting the excluded soil variables in SP1 (sand and AWC).

For SPRS2 data set, the same soil variables detected in the SP2 (sand, AWC and BD) and remote sensing parameters identified in SPRS1 were discarded because of multicollinearity or collinearity with other variables.

MLR analysis was performed considering soil aggregate stability as the dependent variable (MWDmean) and all other factors as independent variables. The results of the MLR model were summarized in Tables 4 and 5. However, each data set was treated into two steps:

Step 1 all selected variables in the preceding paragraph (without collinearity) were used to predict the soil aggregate stability index (MWDmean).

Step 2 significance test (p-value) was performed to detect the least significant variable at the 95% confidence level. Also, the smaller the p-value, the stronger the evidence against the null hypothesis (Kyriacou, 2016). Therefore, the model was developed using variables that are statistically "significant" (Kubinyi, 1996).

The information in Table 4 allows us to confirm that all used variables have not shown any signs of collinearity in our multiple linear regression models. Variance inflation factor (VIF) values were less than 10 (VIF <10) for all data sets variables and ranged between 1.015 and 1.650.

Generally, two main results deserve to be highlighted:

1: Based on the 10-fold cross-validation results, model accuracy was decreased for SP2 and SPRS2 data sets, with an R^2^ of 0.35 and 0.36, respectively (Table 5). Therefore, results were satisfactory for SP1 and SPRS1 data sets with an R^2^ high than 0.5 (acceptable predictive ability) for both data sets, and the RMSE values ranged from 0.277 to 0.401 for all models. Results indicate that the MLR model was more appropriate for the SP1 and SPRS1 data sets than others.

2: Based on the information listed in Table 4, pH, silt, BD, CEC, and remote sensing indices used in Step 1 (NDVI and GVI) were excluded in Step 2 because they had no significant weight in the development of the MLR model for any of the four data sets. These results show that OM and clay were the main predictors (Step 2), and the addition of remote sensing parameters or any other soil properties had no considerable effect on the prediction accuracy.

SP1 and SPRS1 (Step 2) had the same predictors with identical coefficients and an R^2^ of (0.59–0.52 acceptable predictive ability). The same has been observed in SP2 and SPRS2 data sets result with an equal R^2^ of (0.35–0.36 unacceptable predictive ability).

Therefore, based on the best results, Eq. (4) can be used to predict the soil aggregate stability:

(4) *MWDmean = 0.577 + 0.176∗OM + 0.012∗Clay*

**Table 4:** Stepwise multiple linear regression analysis for the 4 data sets.

	Parameter	β	p-value (Sig.)	VIF
	SP1 data set			
Step 1	Intercept	-0,536	0,550	
	pH	0,089	0,322	1,086
	OM	0,172	0,000	1,329
	Clay	0,013	0,000	1,281
	Silt	0,002	0,527	1,403
	BD	0,157	0,533	1,554
	CEC	0,004	0,755	1,341
Step 2	Intercept	0,577	0,000	
	OM	0,176	0,000	1,015
	Clay	0,012	0,000	1,015
	SP2 data set			
Step 1	Intercept	0,053	0,953	
	pH	0,047	0,660	1,044
	OM	0,157	0,000	1,255
	Clay	0,011	0,001	1,224
	Silt	0,005	0,080	1,185
	CEC	0,002	0,726	1,267
Step 2	Intercept	0,673	0,000	
	OM	0,171	0,000	1,026
	Clay	0,009	0,003	1,026
	SPRS1 data set			
Step 1	Intercept	-0,579	0,578	
	pH	0,093	0,324	1,176
	OM	0,175	0,000	1,396
	Clay	0,014	0,000	1,321
	Silt	0,002	0,532	1,403
	BD	0,203	0,440	1,650
	CEC	0,003	0,842	1,372
	GVI	-0,903	0,651	1,133
	NDVI	0,275	0,500	1,258
Step 2	Intercept	0,577	0,000	
	OM	0,176	0,000	1,015
	Clay	0,012	0,000	1,015
	SPRS2 data set			
Step 1	Intercept	0,194	0,851	
	pH	0,039	0,725	1,088
	OM	0,155	0,000	1,284
	Clay	0,011	0,001	1,228
	Silt	0,005	0,078	1,217
	CEC	0,003	0,667	1,349
	GVI	-0,134	0,953	1,05
	NDVI	-0,191	0,687	1,129
Step 2	Intercept	0,673	0,000	
	OM	0,171	0,000	1,026
	Clay	0,009	0,003	1,026

SP1: Soil Properties for 77 samples; SP2: Soil Properties for 114 samples; SPRS1: Soil Properties & Remote Sensing for 77 samples; SPRS2: Soil Properties Remote Sensing for 114 samples; *Β*: coefficient; Sig.: significance and VIF: Variance Inflation Factor.

**Table 5:** Multiple linear regression (MLR) and Random Forest (RF) performances for the MWD prediction.

	MLR		RF	
	R^2^cv	RMSEcv	R^2^cv	RMSEcv
SP1	0.59	0.277	0.6	0.261
SP2	0.35	0.389	0.36	0.397
SPRS1	0.52	0.299	0.57	0.291
SPRS2	0.36	0.401	0.34	0.410

R^2^: coefficient of determination; RMS: root mean square error and cv: cross-validation.

### 3.3 Random forest performance

The RF model's performance was evaluated for each data set by calculating the R^2^, and the root means square error (RMSE) for 10-fold cross-validation. Table 5 shows the results of the four RFs (SP1, SP2, SPRS1 and SPRS2). The value of R^2^ for SP1 and SPRS1 was between 0.57 and 0.6 (acceptable predictive ability), and ranged from 0.34 to 0.36 (unacceptable predictive ability) for SP2 and SPRS2, with low RMSE values for all models (ranged from 0.261 to 0.410).

**Figure 9:**
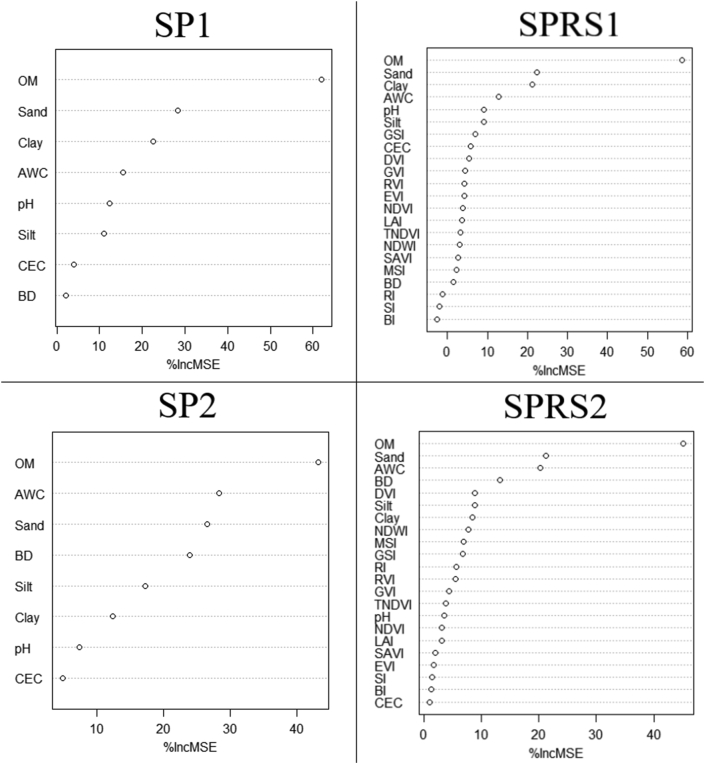
Variable importance rankings of the four Random Forest model. (% IncMSE: percent increase in Mean Square Error; SP1: Soil Properties for 77 samples; SP2: Soil Properties for 114 samples; SPRS1: Soil Properties & Remote Sensing for 77 samples and SPRS2: Soil Properties Remote Sensing for 114 samples).

Figure 9 shows the importance order of variables used as predictors in RF models. Generally, the RF model estimates the importance of variables based on model accuracy variation if one or more variables are removed while keeping the good predictor variables essential for the model (Prasad et al., 2006). Therefore, the most relevant variables for SP1 and SPRS1 are OM, sand and clay. For SP2 and SPRS2, the most important variables are OM, Sand and AWC.

### 3.4 Spatial prediction of MWD

For MWD mapping across the three watersheds, the additional sites from the BR08 dataset were used. As there were differences in the MWD methodology to the SP1 dataset, the RF model was used to estimate the new MWD values of the BR08 data set.

**Figure 10:**
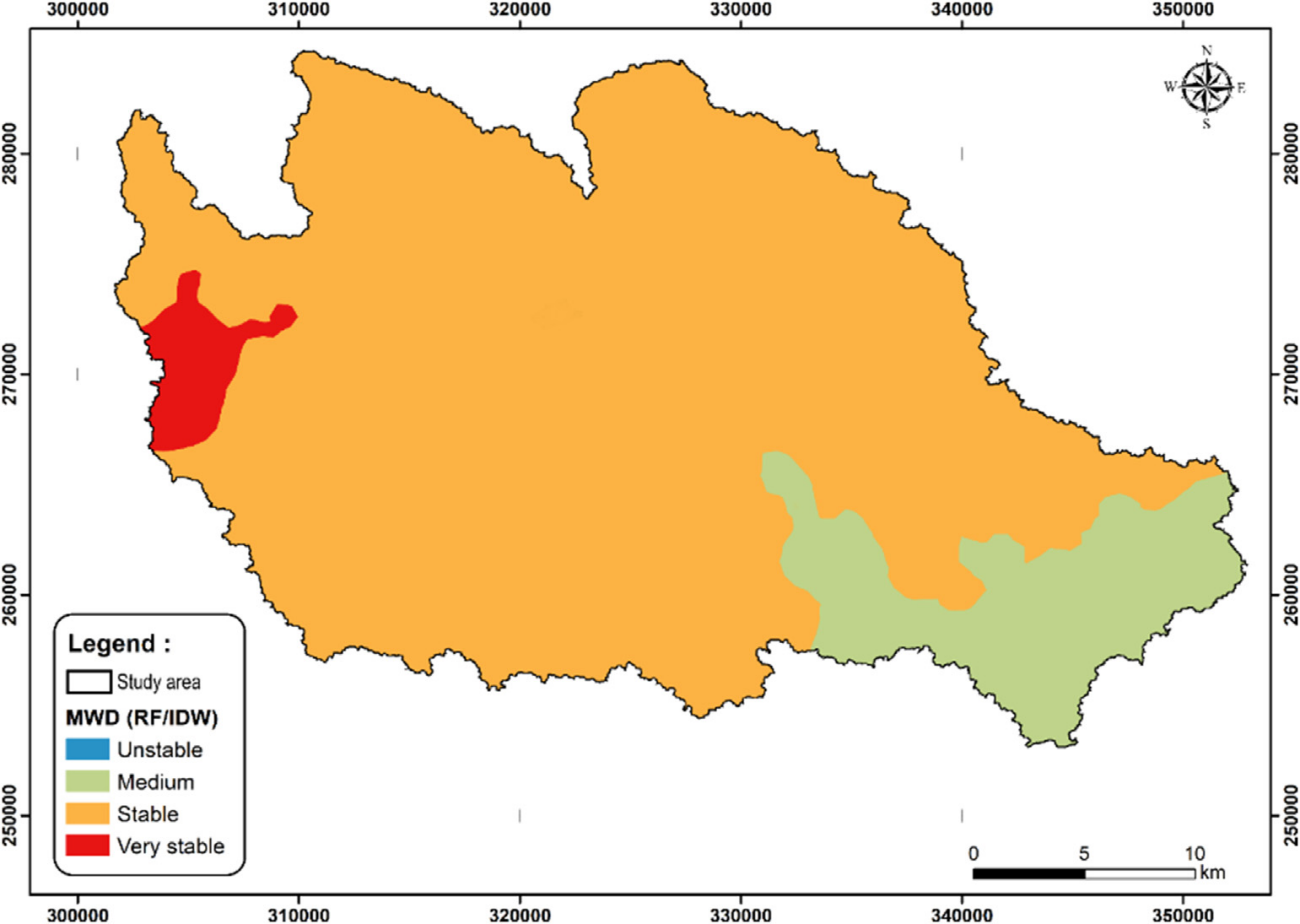
Spatial distribution of soil aggregate stability.

The MWD was mapped for the watersheds using the Inverse Distance Weighting (IDW) method for the 114 samples (77 measured and 37 estimated). The IDW method has shown its capability in soil mapping, and it has been used in several studies worldwide (Chen et al., 2017; Robinson and Metternicht, 2006; Zhang et al., 2011). The values inferred at non-sampled areas by IDW are estimated using a linear combination of values at the sampled places, weighted by an inverse function of the distance from the point of interest to the sample points (Silva et al., 2017). The weights (λ_i_) are expressed in Eq. (5):

(5) λi=1dip∑i=1n1dip

where *di* is the distance between two points, *p* is a power parameter, and *n* represents the number of sampled points used for the estimation. Concerning the created map (Figure 10), the lowest RMSE value (0.289) was obtained using a *p* = 1.5 with a number of neighbors between 10 and 15.

The generated map (Figure 10) using the IDW method shows that the "stable soil" category occupies most of the study area, a small area of the "medium soil" located in the southeastern portion of the study area and the existence of very stable soils in the west part.

**Figure 11:**
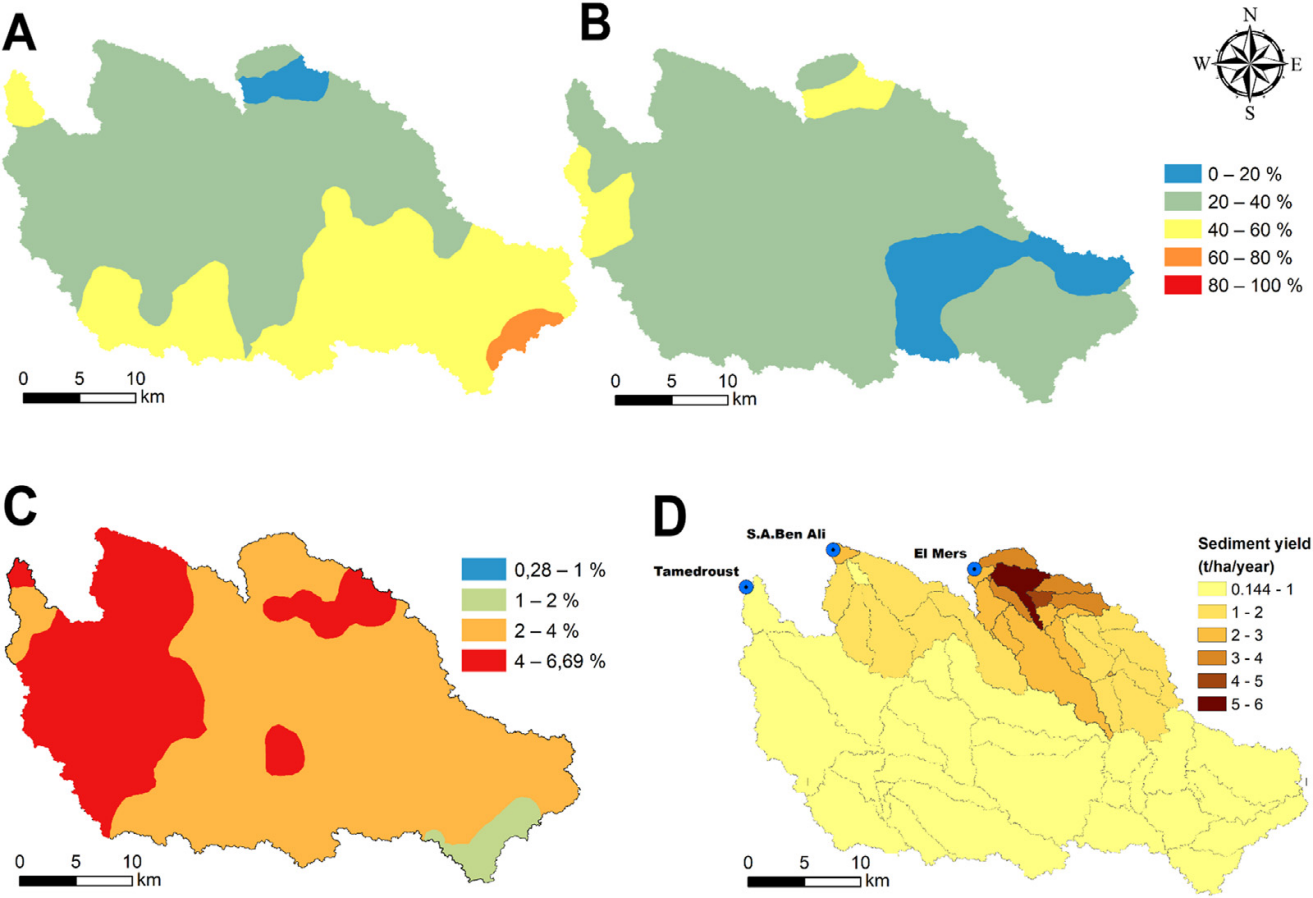
Spatial distribution of A: Sand (%), B: Clay (%), C: organic matter (%) and D: soil erosion rates (t/ha/year).

These results can be explained by returning to the geological features, soil maps and the different soil characteristics. The presence of medium stable soil in the southeastern part can be explained firstly by the geological nature of this part due to the presence of Lutetian formations in the form of siliceous earth; secondly, the presence of shallow soils (Rankers) and Xerosols, which are generally characterized by low levels of organic matter (Figure 11-C). Also, soil analysis results indicate the presence of small or medium quantities of organic matter and a significant presence of sand (between 40 and 60% or higher) (Figure 11-A).

The presence of vertisols and quaternary formations in the western part of the Tamedroust watershed, plus the existence of a gentle slope in the same area, can help provide a favorable context for the development of clayey soils rich in organic matter (Figure 11B and C). This proposition can explain the existence of very stable soils in this part with a significant percentage of clay (between 40 and 60%) and very high rates of organic matter.

In previous studies carried out in the same region (Bouslihim, 2020; Bouslihim et al., 2019, 2020), the authors used the agro-hydrological model SWAT (Soil and Water Assessment Tool) to estimate the rate of erosion in the three watersheds (Tamedroust, Mazer and El Himer). These works showed that all studied watersheds present a weak amount of soil erosion rate, except the downstream part of EL Himer watershed (Figure 11-D), with a high erosion rate compared to other sub-watersheds (with a maximum of 5.20 t/ha/year).

In general, the current study results confirm the low soil erosion rates obtained from the SWAT model in the three watersheds and reported in these previous studies (Bouslihim, 2020; Bouslihim et al., 2019, 2020). That is mainly due to several factors. The most important of which are: (i) soil properties, so that the stable soil occupies most of the study area with a significant percentage of OM and clay, (ii) the low slop values of all watersheds except for the north part of El Himer watershed, and (iii) the scarcity of precipitation as one of the main factors of the soil erosion process, especially in arid and semi-arid regions.

The study results confirm the significant role of organic matter and clay in soils' structural stability (Amezketa, 1999; Annabi et al., 2017; Chaney and Swift, 1984; Chenu et al., 2000; Kavdir et al., 2004). Other studies have shown that some parameters, such as soil microorganisms and their activities and cations (Ca^2+^ and Fe^2+^, among others), are also involved in soil aggregation and stabilization (Lynch and Bragg, 1985; Wuddivira and Camps-Roach, 2007).

### 3.5 Comparison between MLR and RF

Both the MLR and RF methods were acceptable in predicting soil aggregate stability (MWDmean) based on soil properties (SP1) with or without other remote sensing parameters (SPRS1). However, combining this data with the supplementary data (SP2 and SPRS2) decreases the model performance. These results may be explained by variations in data properties, considering that SP1 and BR08 data sets do not have the same source and do not show the same properties and relations between variables, which can be the principal cause of these results. Unlike the significant correlation between MWDmean, Clay and OM of the SP1 data set (77 samples), Pearson's correlation values between variables for the other 37 samples are not significant, with a value of 0.283 between MWD and OM, and -0.264 between MWD and clay, which may reduce MLR model performance.

Thus far, few studies have used the MLR method to predict soil aggregate stability (MWDmean), and none have used Random Forest. Overall, results obtained in this study using MLR to predict MWDmean were lower than those of Marashi et al. (2017). They evaluate the capabilities of MLR and ANNs (in the East of Azerbaijan) for estimating the MWD from two different data sets, routine soil properties (P1) and combination of routine soil properties and fractal dimension of aggregates (P2) data sets (n = 85 samples). The obtained values of R^2^ for the MLR model were 0.78 and 0.90 for P1 and P2, respectively. These results also show that the ANN model was more accurate than the MLR model. Besalatpour et al. (2013) used four different models: inference system (ANFIS), generalized linear model (GLM), ANNs and MLR to predict the MWDmean in a highly mountainous watershed in Iran (n = 160 samples), and found lower values than in the current study. The results obtained for the MLR model ranged from 0.07 and 0.18 for three different sets (soil data, vegetation and topographic data, and the combination between the three covariates). In the same way, Asadi and Bagheri (2010) tried to predict soil aggregate stability with ANNs and MLR models (n = 100 samples) in Iran. The obtained R^2^ values for the MLR model ranged from 0.15 to 0.39, which is lower compared to the results obtained in the current study.

The RF method showed varying results when it was used to predict different soil properties. In a study in Denmark, Pouladi et al. (2019) compared the performance of four machine learning techniques (kriging, Cubist, Random Forest and regression-kriging) to predict soil organic matter using different environmental predictors for 285 soil samples. The value obtained of R^2^ for the RF technique was 0.89, with an RMSE of 4.2. In another study in South India (Dharumarajan et al., 2017), used the RF technique (116 samples) to predict three soil properties and reported lower R^2^values for organic carbon (0.23) and pH (0.3) and a satisfactory value for electrical conductivity (0.62). Chagas et al. (2016) evaluated the efficiency of using remote sensing data based on MLR and RF to predict the sand, silt and clay contents for 399 samples. They reported similar results between the two methods, with satisfactory results for sand (0.47–0.51) and clay (0.48–0.49), and lower values for silt (0.08–0.2). These previous studies show that the results of the RF are varied and related to many factors such as the size of the data set, the scale of variation, and also the relations between dependent and independent variables, which may be the same reason for the results achieved during this current study.

According to the literature, one of the main advantages of the RF model is that it estimates each variable's relative importance in the model, unlike MLR, which keeps only the highly correlated variables due to the stepwise selection. On the other hand, the RF avoids removing predictive variables that may be important to prediction, even if correlations exist between them (collinearity) (Akpa et al., 2014; Cutler et al., 2007).

## 4 Conclusion

We tested two completely different models (MLR and RF) to predict soil aggregate stability, which can be considered an essential indicator for monitoring soil quality, but that requires considerable time and effort. Therefore, the development of models was performed using several soil paraméters and remote sensing indices. Overall, both models have performed acceptably in predicting soil aggregate stability (MWDmean) based on soil properties, with or without other remote sensing indices. However, the combination of SP1 and BR08 decreases both model performances, which was maybe explained by variations in soil data properties for both data sets. Thus, the addition of remote sensing indices to soil properties does not improve results. One cannot yet judge the best model based on these results. Therefore, the sample size from the same source must be increased to ensure more excellent uniformity of sampling and analysis, which could help create a better recognized and understood process of predicting soil aggregate stability. Finally, the lack of some previous research studies limited the possibility to discuss some of the results of this manuscript. However, the results obtained in this study are generally satisfactory.

## Declarations

### Author contribution statement

Y. Bouslihim: Conceived and designed the experiments; Performed the experiments; Analyzed and interpreted the data; Contributed reagents, materials, analysis tools or data; Wrote the paper.

A. Rochdi, N. El AVmrani Paaza: Conceived and designed the experiments; Performed the experiments; Analyzed and interpreted the data.

### Funding statement

This research did not receive any specific grant from funding agencies in the public, commercial, or not-for-profit sectors.

### Data availability statement

Data will be made available on request.

### Declaration of interests statement

The authors declare no conflict of interest.

### Additional information

This paper is dedicated to the memory of Dr. Yves Le Bissonnais.
